# 26600 HiTech and Health Interest Trends in Rural Neighborhoods are Associated with COVID-19

**DOI:** 10.1017/cts.2021.518

**Published:** 2021-03-30

**Authors:** Melody Greer

**Affiliations:** University of Arkansas for Medical Sciences

## Abstract

ABSTRACT IMPACT: Current, complete, unbiased, and accurate information, which includes patient social and environmental context, is necessary to understand health outcomes. OBJECTIVES/GOALS: Literacy in technology empowers patients in improving their health. We hypothesize that by integrating this information into clinical information, obfuscated relationships may become apparent. To test this, we have combined technological literacy elements with clinical data and test results from patients at risk for severe COVID-19 reactions. METHODS/STUDY POPULATION: Zip level data was appended to approximately 55,000 clinical records of COVID-19 positive and negative patients with comorbidities linked to high illness severity (e.g., diabetes, heart disease). The patient zip code was matched to zip code level health and technology interest indicators. Health interest indicates the level of interest in health such as research, exercising, better dieting, preventive care, etc., and is ranked from 0 to 9. The technology interest indicator is a binary flag indicating technology adopters. These lifestyle factor data points were obtained from survey data and purchasing patterns using transactional and response information from self-reported sources. For each zip code, the index values were represented by a percentage of that population. RESULTS/ANTICIPATED RESULTS: There is a pronounced difference between urban and rural areas with respect to interest in health and technology. In neighborhoods where most residents are interested in both health and technology, the percentage of COVID-19 cases was smaller. A Wilcoxon signed-rank test indicated that the distributions were statistically different (p-value = 4.606e-06) when evaluating the low interest values for health and technology, and (p-value = 1.069e-09) when there was high interest in health and technology against COVID-19 results. In addition, the health and technology indicator variables are not correlated with income at the zip code level. At the low index values, interest in health and technology, the correlation was -0.0856, and at the high end, the correlation was -0.0436. DISCUSSION/SIGNIFICANCE OF FINDINGS: This result is significant for COVID-19 research because it describes a methodology for identifying patients who may be at higher risk for contracting the disease. This relationship was reflected in electronic health record data only after zip-level information was added. Moreover, this was true at across income levels.

